# Comparison of conventional preparation with modified access preparation on fracture resistance of primary molars - A finite element analysis

**DOI:** 10.1016/j.jobcr.2023.08.010

**Published:** 2023-09-06

**Authors:** Harshini Nivetha Easwaran, Kavitha Swaminathan, Selvakumar Haridoss, M.S. Muthu, Priya Jayakumar

**Affiliations:** a Department of Pediatric and Preventive Dentistry, Sri Ramachandra Dental College and Hospital, Sri Ramachandra Institute of Higher Education and Research, Chennai, India; b Centre for Early Childhood Caries Research, Department of Pediatric and Preventive Dentistry, Sri Ramachandra Dental College and Hospital, Sri Ramachandra Institute of Higher Education and Research, Chennai, India; c Centre of Medical and Bio-Allied Health Sciences Research, Ajman University, Ajman, United Arab Emirates

**Keywords:** Access preparation, Conservative endodontics, Fracture resistance, Minimally invasive endodontics, Pediatric endodontics, Finite element analysis

## Abstract

**Objective:**

To investigate the influence of Conventional Straight (CS) line and Modified Straight (MS) line access preparations with various restorative materials on the fracture resistance of primary molars using Finite Element Analysis (FEA).

**Methodology:**

Three FEA models for each of the primary molars were divided into Group I- Intact tooth model; Group II- Model with CS outline and Group III- Model with MS outline. Based on the restorative material used, Group II and III were further subdivided into subgroup 1- GIC restoration, subgroup 2 – composite resin with GIC base and subgroup 3- Stainless Steel Crown (SSC). Each model was subjected to 5 different force loads directed at the occlusal surface. Maximal von Mises (VM) stresses calculated from stress distribution patterns.

**Result:**

The maximum displacement, in all the models of primary molars were seen in GIC restored models in molars with both CS and MS access whereas the minimal displacement was seen in the SSC restored molars of MS group.

**Conclusion:**

In primary maxillary second molar and mandibular first and second molar with intact marginal ridges, the fracture resistance of tooth with MS outline restored with GIC base followed by Composite resin was comparable with the tooth restored with SSC and CS outline.

**Clinical outcome:**

Based on the results of this FEA analysis, composite restorations with MS outline would be appropriate for endodontically treated primary molars that have intact margins.

## Graphical abstract

**Figure:**
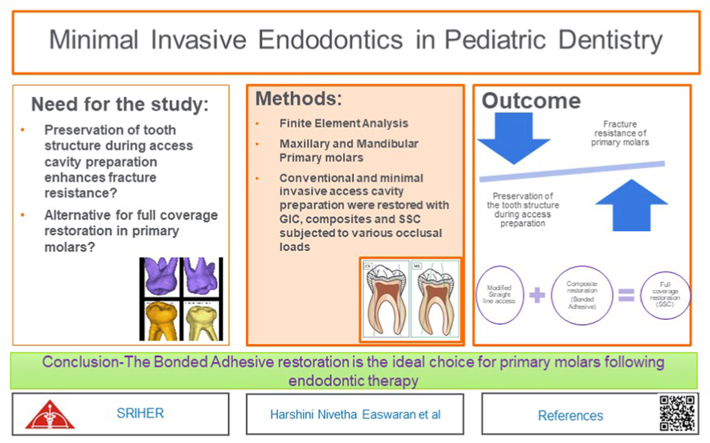

## 1 Introduction

The long-term success of endodontically treated teeth is still a great challenge because of their reduced fracture resistance. Extensive removal of dental tissue could have a negative impact on the integrity of the dental structure, facilitating fracture.^1^ To overcome this, Minimally Invasive Endodontics (MIE) and Conservative Endodontic Cavities (CEC) have been advocated for the treatment of permanent teeth.^2^ These procedures improve endodontic treatment by designing precise access cavities and pulp-chamber finishing while preserving as much tooth structure as possible.^3^ The influence of various endodontic access cavities on fracture resistance has been studied in detail in the permanent dentition.^4^ Several Studies reported no differences pertaining to the fracture resistance of teeth with MIE and the conventional straight-line (CS) access preparation in the permanent teeth,^5^^,^^6^ while a few studies found significant differences between MIE and CS.^7^, ^8^, ^9^ These studies had a few shortcomings, such as a lack of anatomical matching, lack of consideration of the volume of the pulp chamber and the thickness, height, and/or volume of the remaining tooth tissue, and lack of studies on the impact of the functional and parafunctional forces that influence the fracture resistance. The use of Finite Element Analysis (FEA), a complementary tool for solving dental biomechanical problems by matching samples based on the age and 3D morphological dimensions of teeth, and also simulating oral masticatory function and aging using mechanical and/or thermal cycling to determine the fracture resistance.^6^

Clinical trials, systematic reviews, and meta-analyses have established the efficacy of stainless steel crowns (SSC) as a semi-permanent restorative therapy for endodontically managed primary teeth.^10^ Despite these, however, there is a paucity of evidence on the durability of SSC pertaining to fracture resistance. A FEA analysis of teeth restored with SSC would provide valuable data on the physical response of the crown-tooth system to masticatory stresses. Mannocci et al. (2009) reported composite resin as a superior restorative material in endodontically treated permanent teeth with CEC.^11^ However, to the best of our knowledge, the influence of MIE and CEC under the application of various masticatory forces has not yet been studied in the primary teeth. Understanding the influence of CEC on fracture resistance can pave the way for the use of a permanent restorations other than SSC that would preserve the integrity of primary dentition until the eruption of their permanent successors. Therefore, the objective of this study was to use FEA to investigate the influence of CS and modified straight-line (MS) (a type of CEC) endodontic access cavities with various restorative materials on the fracture resistance of endodontically managed primary molar.

## 2 Materials and methods

Ethical approval for this study was obtained from the Institutional Ethics Committee of Sri Ramachandra Institute of Higher Education and Research (Ref. No. CSP/21/OCT/100/530).

## 3 Description of the FEA model generation

**Fig. 1:**
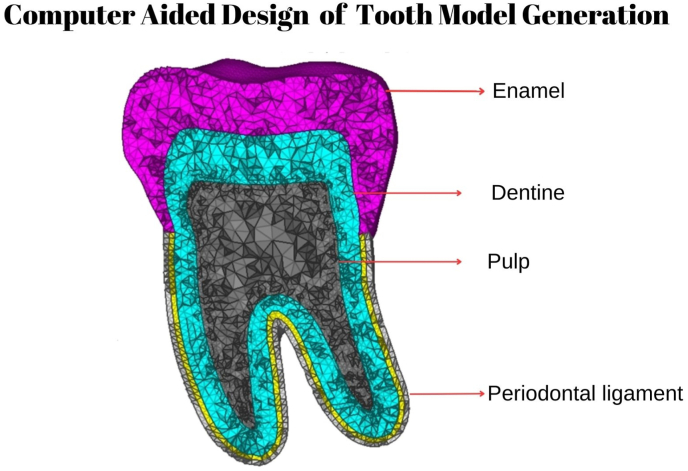
Finite element model of primary mandibular second molar.

In this study, FEA generated tooth models of primary maxillary and mandibular first and second molars have been analyzed. A Cone Beam Computed Tomographic (CBCT) image of intact caries-free, primary maxillary and mandibular first and second molars were extracted separately from the CBCT images. These images were then modeled as a 3-Dimensional (3D) model, including the properties of enamel, dentin, dental pulp, and cementum using MIMICS 16.0; Materialise, Leuven, Belgium software [Fig. 1]. The material properties (elastic modulus and Poisson ratio) are presented in Table 1. The periodontal ligaments surrounding the roots and the bones were established. The endodontic access cavities were then designed on the individual tooth models with ANSYS Inc, Canonsburg, PA Software.

**Table 1:** The mechanical properties of the investigated materials.

Component	Young's Modulus (GPa)	Poisson Ratio
Enamel	80.35	0.33
Dentin	19.89	0.31
Pulp	2	0.45
Periodontal ligament	50	0.45
Cortical bone	13.7	0.3
Cancellous bone	13.7	0.3
Zinc Oxide Eugenol	5.4	0.35
Composite	13.46	0.31
Stainless Steel Crown (SSC)	180	0.3
GIC	12.0	0.3

## 4 Cavity design

Three 3D models for each of the primary maxillary and mandibular molars were generated and grouped as follows (i) Group I- the intact teeth model, (ii) Group II - Conventional model which involved Conventional Straight line access cavity preparation and, (iii) Group III – Conservative Endodontic Cavity model which included Modified Straight line access cavity preparation. The two access cavity designs were derived based on the anatomic landmarks inspired by the studies of Eaton et al. and Lin et al. with slight modifications to accommodate deciduous teeth.

**Fig. 2:**
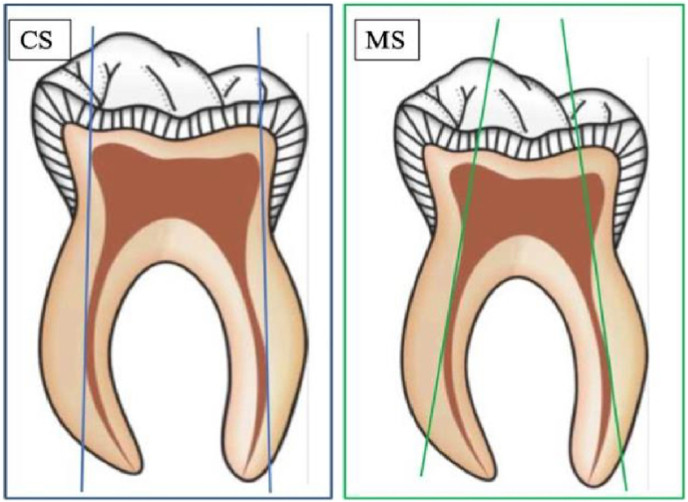
Schematic representation of conventional straight line and modified straight line access cavity.

CS access was determined by a line drawn parallel to the outer surface of the pulp chamber to the primary canal curve in the view of maximum curvature. MS outline was determined by a line drawn from the outer canal surface at the primary canal curve through the outer canal surface at the level of furcation.^12^^,^^13^ The illustrations of the CS and MS outlines are represented in Fig. 2.

In total, 28 models were generated: (i) four intact primary maxillary and mandibular first and second molars in the control group (Group I); (ii) 12 models in the CS group (Group II), comprised of four models each for the subgroup 1- Glass Ionomer Cements (GIC), subgroup 2- GIC and Composite and subgroup 3 -Stainless Steel Crowns (SSC) and (iii) 12 models in the MS group (Group III), comprised of four models each for the subgroups 1 -GIC, subgroup 2- GIC and Composite and subgroup 3 –SSC.

**Fig. 3:**
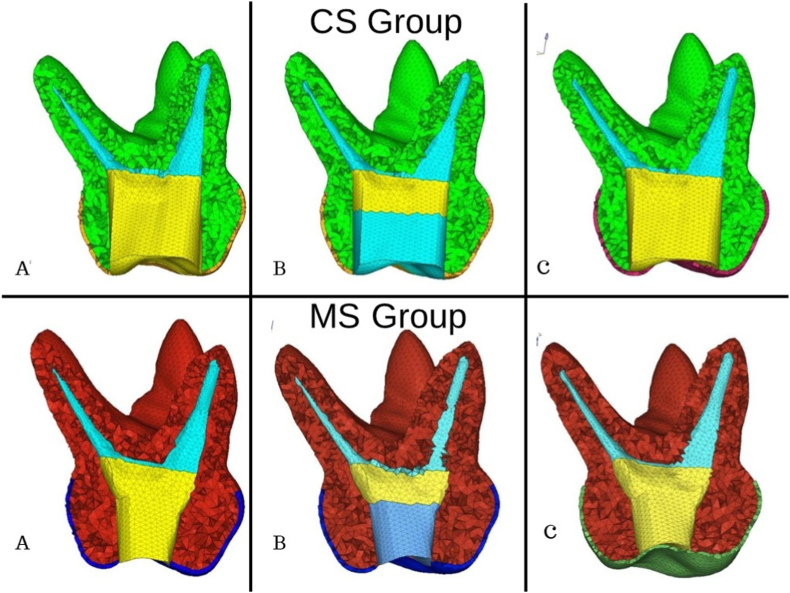
Different access cavity designs of primary maxillary first molar restored with A) GIC B) GIC & composite C) SSC.

For all the generated samples (primary maxillary and mandibular first and second molars), one intact 3D model for the control group was generated. Three models were then generated with CS access outline. The first model (subgroup 1) was restored with the properties of GIC up to the occlusal surface, the second model (subgroup 2) was restored with properties of GIC base up to 2 mm from the level of canal orifice and the properties of composite from the GIC base up to the occlusal surface, and the third model (subgroup 3) was restored with properties of SSC. Similarly, three models were generated with MS access outline as mentioned above. In the endodontic treatment models, the teeth were prepared endodontically and the canals were filled with Zinc Oxide Eugenol [Fig. 3].

## 5 FEA analysis

The teeth models in all 3 groups, (Group I, II and III) were subjected to a series of loads at 0°, 45° and at 90° to simulate vertical, lateral mastication load and maximum masticatory force respectively.^14^ Forces of 220 N, 246 N, and 275 N were applied to simulate the minimum, mean and maximum force in the primary dentition and loads of 311 N and 396 N were applied to simulate mean and maximum force in the mixed dentition.^15^ These forces were applied at 5 different occlusal contact points on the teeth models, as the pressure load to simulate the maximum mastication force.^14^

After the application of specified load to the teeth models, the stress patterns, the stress distribution, and the displacement were analyzed. The resultant Von Mises (VM) stress, a value to determine the fracture resistance and displacement of the restorative materials for the given stress is represented as a color-coded figure.

## 6 Results

**Fig. 4:**
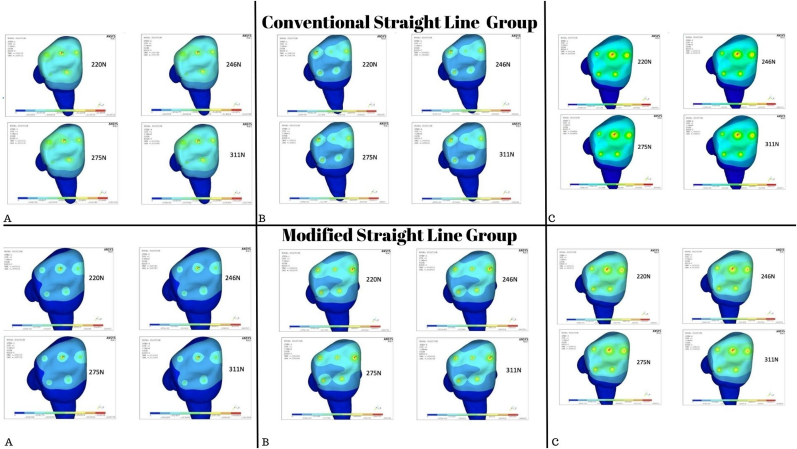
Deformation of primary mandibular first molar under different loading conditions restored with A) GIC B) GIC & composite C) SSC.

The images obtained from the Finite Element Analysis are all color graded such that dark blue represents areas experiencing minimal von Mises stress and red represents areas experiencing maximal von Mises stress. Forces of 220 N, 246 N, 275 N, 311 N, and 396 N were applied at angles of 0°, 45°, and 90° in all 28 of the tooth models generated. The VM stress and the displacement were analyzed individually for all models [ Fig. 4]. The peak VM stress occurred at the site of load application in all the generated models.

## 7 Primary maxillary first molars

**Fig. 5:**
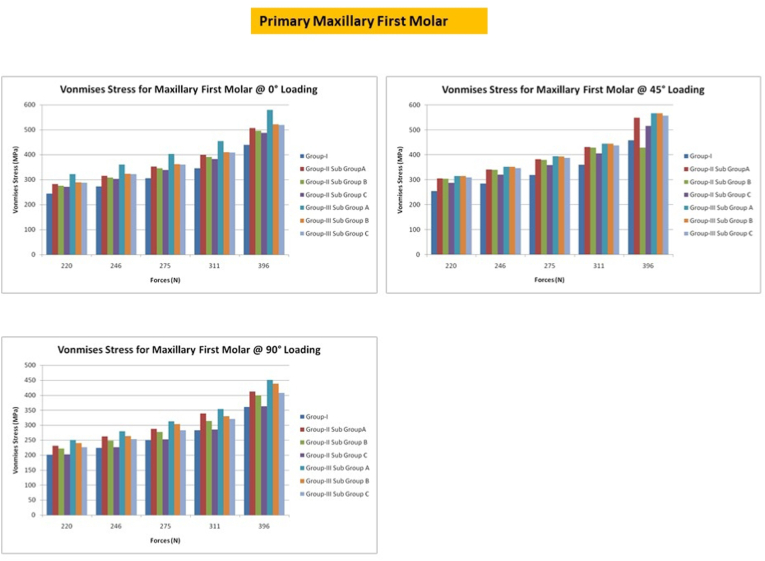
Von misses stress in primary first maxillary molar under different loading conditions.

Under application of the mentioned forces at an angle of 0° to simulate the lateral masticatory load, the maximum stress occurred along the marginal ridges of the CS Access with a higher deformation (0.0302), followed by MS access with a deformation of 0.0173. Forces directed at an angle of 0° in CS access caused propagation of stresses along the buccal and lingual surfaces. The application of stress at 45° caused the distribution of stress to the buccal aspect of the tooth. The deformation was higher in CS access with GIC (0.02656) followed by MS access with GIC (0.0087). When the forces were applied at an angle of 90° to simulate the maximum masticatory force, the peak VM stress occurs at the margins in the CS access and MS access [Fig. 5].

## 8 Primary maxillary second molars

**Fig. 6:**
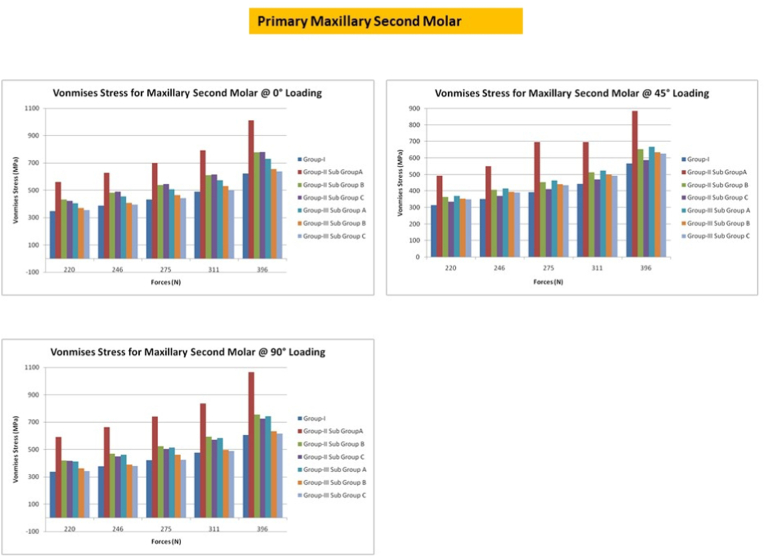
Von misses stress in primary second maxillary molar under different loading conditions.

With a force direction of 0°, the MS approach with SSC showed the highest fracture resistance, whereas the CS approach with GIC showed the lowest fracture resistance. When the direction of force was at an angle of 45°, the highest VM stress was observed in CS access with GIC, followed by MS access with GIC. At an angle of 90°, the maximum stress was concentrated on the mesial inclinations of the restoration and the tooth interface [Fig. 6].

## 9 Primary mandibular first molars

**Fig. 7:**
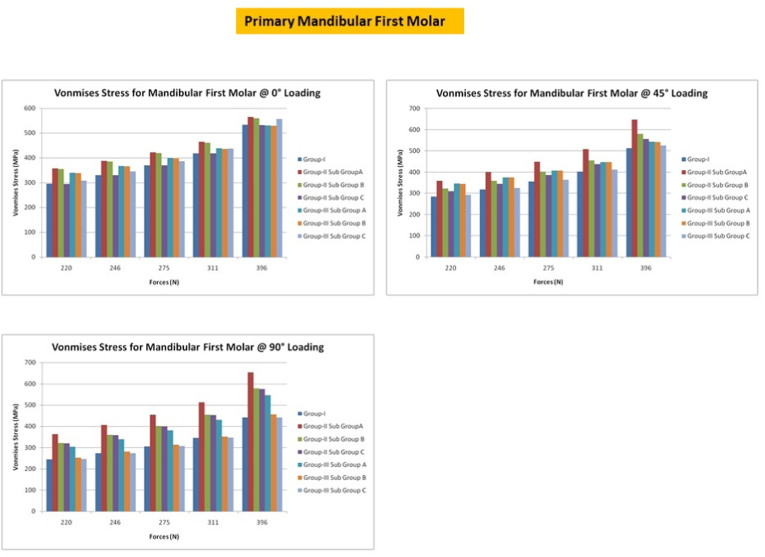
Von misses stress in primary first mandibular molar under different loading conditions.

A CS access with SSC exhibited the greatest fracture resistance when forces were directed at an angle of 0°, whereas a CS access with GIC exhibited the least fracture resistance. MS and CS access with SSC had equivalent fracture resistance. Under 45° loads, the peak VM stress occurred along the buccal and lingual inclines of the CS access, and along the buccal inclines of the control group and MS access with GIC. In MS access with GIC, VM stress was distributed along the distobuccal cusp with forces directed at 90°. Peak stresses were found at the distobuccal cusp in control group and MS access [Fig. 7].

## 10 Primary mandibular second molars

**Fig. 8:**
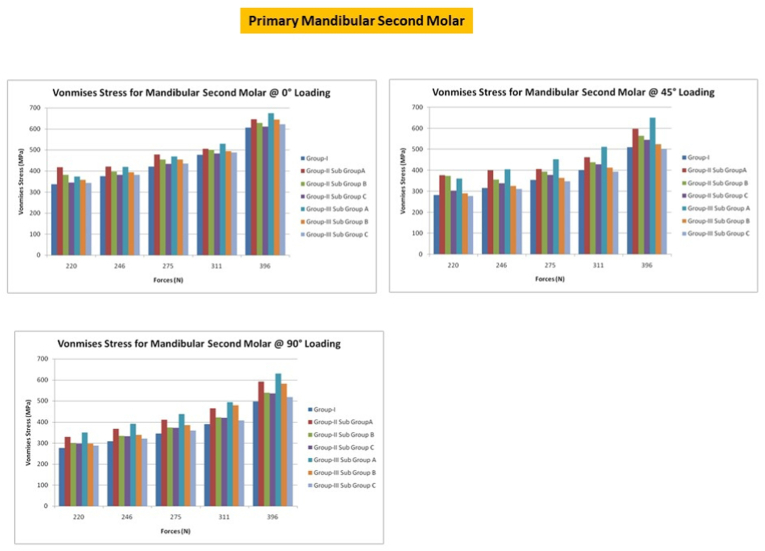
Von misses stress in primary second mandibular molar under different loading conditions.

With forces directed at 0°, peak VM stress occurred in CS access with GIC along with the point of load application in the buccal inclines, while the peak VM stress in Control group and MS access occurred at the site of load application. At 45° loads, fracture resistance in Composite with MS access was higher than that in CS access with SSC. Under 90° loads, peak VM stress CS access with GIC followed by MS access with GIC. Within CS access, it could be observed that the fracture resistance of Composite restored group was comparable with that of SSC. In all the primary molars, it could be observed that the maximum deformation occurred in the conventional group followed by the conservative group [Fig. 8].

## 11 Discussion

Conventional access preparations have consistently achieved the required goals, concerns were raised on their effect on tooth survival and ability to resist fracture.^16^ Access cavity designs involving minimal removal of tooth tissue for gaining entry to pulp chambers have been described for better fracture resistance during root canal treatment. MIE emphasizes preservation of a maximal amount of healthy tooth tissue, which could be implemented in the decision-making process of the design of an endodontic access cavity, and the preparation of the root canals or the restorations.^17^ With the rapid development of technology, particularly, with dental operating microscopy and CBCT imaging, MIE treatment should become an inevitable option in the near future.^18^ One of the most important conditions that contribute to the susceptibility of a tooth to fracture includes the removal of large amounts of sound dentine during the endodontic and restorative procedures.^19^

Tooth fractures are closely related to stress distribution. However, it is difficult to measure stresses in a tooth that has a complex shape situated in an intricate environment using a measuring device.^20^ FEA has been accepted as a non-invasive tool for studying biomechanics and the influence of mechanical forces on biological systems.^21^ Consequently, the results of our study may be practically applicable in the clinical setting.

In our study, we used loads of 220 N, 245 N, 275 N, 311 N and 396 N to simulate masticatory loads. A meta-analysis by Jayakumar P et al., 2023 concluded that 220 N, 245 N and 275 N are the minimum, mean and maximum masticatory forces in the primary dentition. Whereas forces of 275 N, 311 N and 396 N were considered the minimum, mean and maximum masticatory forces in the mixed dentition respectively.^15^ As the primary molars, course through both the primary and mixed dentition stages, these forces were included in the analysis. Loads were applied at an angle of 0°, 45° and 90° to simulate vertical and lateral masticatory force and maximum masticatory force respectively which is in accordance with Jiang et al., 2018.^14^ The load was applied at 5 different sites on the models on the highest cuspal elevations with two points along the buccal cusps, two points at the palatal cusps and one point at the central fissures, to simulate the real chewing situation in which the 5 contact points of the molars experience maximum masticatory stress.^22^

Hegde et al. (2019) reported that the endodontically treated tooth when not suitably restored with a SSC results in fracture of the underlying tooth structure.^23^ The results of our study are in accordance with these studies, as the tooth being restored with SSC in both the CS and MS group exhibited higher fracture resistance. The efficacy of SSC could be explained since it has a greater elastic modulus of 180 GPa than that of the natural tooth which is 80.35 GPa in enamel and 19.89 GPa and is much larger than that of any restorative material used in pediatric dentistry.

Our study suggests that the fracture resistance of an endodontically restored tooth with SSC and CS access cavity design were comparable to the tooth with MS access cavity design which was restored with composite in maxillary and mandibular second molars. However, a study by Singhal et al., 2021 on the assessment of fracture resistance with different access cavity designs on primary mandibular molars reported that CEC was significantly higher than that of the TEC design.^24^

In the present study, the prepared access cavities were restored with bonded resin composite in subgroup 2 under MS and CS groups. However, in our simulated models of primary maxillary and mandibular first molars, it could be observed that the use of composite resin did not improve the fracture resistance in comparison with GIC. These results are in concordance with Sengul et al., 2014 who reported that the restorative materials in primary second molars presented lower stress values than primary first molars.^25^ An FEA study by Bratosin et al., 2014 reported that GIC restored teeth showed greater stress concentration as compared to composite material in deciduous teeth.^26^

This study is unique in that it evaluates the effect of access cavity design upon the fracture resistance of primary teeth under masticatory loads that replicate the occlusal bite forces of primary and mixed dentition. Consequently, the results of our study may have practically applications in the clinical setting.

The limitations of the current study were attributed to the disadvantages of the linear FEA method that could be summarized as follows: (1) FEM models did not include the whole jaw so that the results represented only the response of part of the tooth structure; (2) To ensure consistency, the materials are assumed to be homogenous, isotropic, and linearly elastic, which they are not (3) The change in soft tissue material properties during the craniofacial growth were not considered; (4) The fatigue - induced failure was not taken into consideration in this study. The absence of fatigue loading is not considered.

In clinical conditions, endodontic access cavities need to be sized appropriately and treated minimally to preserve as much tooth structure as possible. The anatomy of the root canal system should be evaluated before conducting minimally invasive cavity preparation. However, further longitudinal studies or randomized controlled trials with robust methodology are required to validate the results.

## 12 Conclusion

The following conclusions can be drawn by using finite element analysis.

• The preservation of tooth structure by conservative access preparation enhances the fracture resistance of the tooth.
• MS access with composite restoration as an alternate treatment option for conventional SSC in primary teeth necessitating pulpectomy with intact marginal ridges based on the findings of this study.

## Presentation(s) or awards at a meeting

Not Applicable.

## Source(s) of support and funding

Self-funded

## Consent to participate

Not Applicable.

## Patients’ consent form

Not Applicable.

## Ethical approval and/or institutional review board (IRB)

Obtained.

## Declaration of competing interest

The authors do not have any financial interest in the companies whose materials are included in this article.
